# Current status and training needs of trainee anesthesiologists in lung transplantation anesthesia in China: A single-center survey

**DOI:** 10.1016/j.heliyon.2022.e12428

**Published:** 2022-12-19

**Authors:** Yan Zhou, Zhong Qin, Guilong Wang, Wenyi Chen, Xin Zhang

**Affiliations:** a Department of Anesthesiology, The Affiliated Wuxi People's Hospital of Nanjing Medical University, Wuxi, Jiangsu, 214023, China; b Department of Anesthesiology, Duke University School of Medicine, Durham, NC, 27710, USA

**Keywords:** Lung transplant, Anesthesia, Training program, Survey, China

## Abstract

**Background:**

Perioperative management involving anesthesiologists plays an important role in prognosis of recipients after lung transplantation. Since the development of lung transplantation, the demand for specialized anesthesiologists continues to increase. As the largest lung transplant center in China, the Wuxi People's Hospital was tasked with trainee anesthesiologists throughout the country in lung transplantation anesthesia. This study aimed to evaluate the current status and training needs of anesthesiologists for the anesthetic management of lung transplantation in Wuxi People's Hospital between 2015 to 2020.

**Methods:**

Overall, 53 trainee anesthesiologists for lung transplantation from 35 hospitals were investigated anonymously in our survey. The questionnaire included the anesthesiologists' demographic information, level of satisfaction, training needs and current status in their hospitals. We divided the doctors into two groups depending on the trainee anesthesiologists’ seniority and professional title: intermediate and senior. Survey data were compared between the groups.

**Results:**

Significantly more doctors in senior-level positions had clinical research experience than did doctors in intermediate-level positions (P = 0.041). All doctors were highly or very highly satisfied with the training received. Doctors in intermediate-level positions preferred training periods of 4–6 months, while those in senior-level positions preferred 1–3 months of training (P = 0.044). Most doctors considered theoretical courses to be lacking (69.0%), followed by a lack of scenario simulation teaching (54.8%). The most desirable programs were transesophageal echocardiography (TEE, 71.4%) and extracorporeal membrane oxygenation (ECMO, 64.3%). ECMO technology was available in the hospitals of 95.2% of respondents; however, only 2.4% of doctors said the anesthesiology department took charge of perioperative ECMO. Significantly more senior-level doctors chose calibrated pulse contour analysis (P = 0.018) and significantly more intermediate-level ones chose TEE (P = 0.049). Disappointingly, 21.4% doctors reported a lack of certification evaluation for trainee anesthesiologists at their hospitals.

**Conclusions:**

Different training programs should be set up according to the trainee anesthesiologists’ level of seniority and training needs. Theoretical courses and scenario simulation training must be added to improve the training program. Moreover, the training of TEE and ECMO requires greater attention. Finally, a standardized completion assessment is required for trainee anesthesiologists.

## 1 Introduction

Lung transplantation is an effective treatment for long-term survival of patients with end-stage lung disease [1]. Recently, lung transplantation has developed rapidly, and the number of transplants has increased year on year. In China, the number of lung transplants in 2018 reached a total of 403 [2]. Wuxi People's Hospital is the largest lung transplant center in China and among the top three globally, performing 150 lung transplants in 2018 [2]. Anesthesiologists play a vital role in cardiothoracic transplantations. Donor management, recipient preoperative evaluation, perioperative management, and postoperative monitoring require the active participation of anesthesiologists [3]. The perioperative management of anesthesiologists has an important impact on prognosis of recipients after lung transplantation [4]. With the development of lung transplantation, the demand for anesthesiologists specialized in lung transplantation has increased. Doctors who study anesthesia for lung transplantation often already have rich clinical experience. Combining their characteristics and training needs to complete the standardized training of anesthesia for lung transplantation in a brief period of time is worth studying in the field of continuing education for anesthesia. As the largest lung transplant center in China, the Wuxi People's Hospital undertook a lot of continuing education in anesthesia for lung transplantation, and were tasked with the training thereof for trainee anesthesiologists throughout country [2]. Improving the overall quality of training, including medical technology, is of great importance to ensure that training programs are scientifically sound and consider the needs and current status of trainee anesthesiologists. Therefore, this study aimed to evaluate the current status and needs of the trainee anesthesiologists who had received training for lung transplantation anesthesia in Wuxi People's Hospital from 2015 to 2021.

## 2 Materials and methods

### 2.1 Survey

The study was approved by the appropriate Institutional Review Board (IRB) of the Affiliated Wuxi People's Hospital of Nanjing Medical University, and written informed consent was obtained from all participants. All Chinese trainee anesthesiologists studying lung transplantation anesthesiology in the Department of Anesthesia of Wuxi People's Hospital from 2015 to 2020 were surveyed anonymously using an online-based questionnaire. The questionnaire was created using a dedicated software (WenJuanXing, owned by Changsha Ranxing Information Technology Co., Ltd., China) and sent by WeChat (Tencent Holdings Limited, 2018. WeChat, Available at: https://www.wechat.com) in December 2021 to the visiting doctors, and a clarification stating the voluntary nature of the survey participation. The questionnaire included 33 questions. Twenty-eight were single-choice and five were multiple-choice questions (seen in Supplemental Material). After the survey, we divided the data into two groups, depending on the trainee anesthesiologists' professional title: doctor in charge (intermediate); and associate senior doctor and senior doctor (senior). Completed surveys were collected anonymously on the internal server of WenJuanXing.

### 2.2 Statistical analysis

All data were analyzed using SPSS v22.0 (IBM SPSS Statistics for Windows, Version 22.0. Armonk, NY: IBM Corp). Data were expressed as numbers (with percentages). Responses to single-choice questions were compared between the two groups using the χ^2^ or Fisher's exact test. Responses to multiple-choice questions were compared between the two groups using the χ^2^ test. A p-value less than 0.05 was considered statistically significant. This was an exploratory analysis; no adjustments were made for multiple comparisons.

## 3 Results

### 3.1 Demographic characteristics and training needs of trainee anesthesiologists

Of a total of 53 trainee anesthesiologists from 38 hospitals, 42 (79.2%) doctors from 35 hospitals responded to the survey. As shown in Table 1, 18 (42.9%) were intermediate-level doctors and 24 (57.1%) were senior-level doctors. Of all trainee anesthesiologists, 31 (73.8%) were men and 11 (26.2%) were women. Senior-level doctors showed significantly different age and years of work in anesthesiology department (P = 0.002). Eleven (45.8%) doctors in senior positions and two (11.1%) doctors in intermediate positions had PhD degrees (P = 0.037). Twelve (66.7%) doctors in intermediate positions and 22 (91.7%) in senior positions had clinical research experience (P = 0.041). Before the training, nine (21.4%) doctors had experience in lung transplantation anesthesia and 39 (92.9%) had experience in cardiac anesthesia, while 11 (26.2%) had no experience with Swan-Ganz catheterization and 18 (42.9%) had no experience with transesophageal echocardiography (TEE).

**Table 1:** Demographic characteristics and training needs of trainee anesthesiologists.

	All doctors (n = 42)	Intermediate (n = 18)	Senior (n = 24)	P-value
Sex (male/female)	31/11	11/7	20/4	0.103
Age (years)				0.002
<30	3 (7.1%)	2 (11.1%)	1 (4.2%)	
30–35	14 (33.3%)	9 (50.0%)	5 (20.8%)	
36–40	14 (33.3%)	7 (38.9%)	7 (29.2%)	
41–45	8 (19.0%)	0 (0.0%)	8 (33.3%)	
>45	3 (7.1%)	0 (0.0%)	3 (12.5%)	
Highest level of education				0.037
Bachelor's Degree	7 (16.7%)	4 (22.2%)	3 (12.5%)	
Master's degree	22 (52.4%)	12 (66.7%)	10 (41.7%)	
PhD Degree	13 (31.0%)	2 (11.1%)	11 (45.8%)	
Years working in anesthesiology department				<0.001
<5	1 (2.4%)	1 (5.6%)	0 (0%)	
5–10	7 (16.7%)	5 (27.8%)	2 (8.3%)	
10–15	13 (31.0%)	10 (55.6%)	3 (12.5%)	
>15	21 (50%)	2 (11.1%)	19 (79.2%)	
Research experience				
None	5 (11.9%)	3 (16.7%)	2 (8.3%)	0.409
Basic research experience	19 (45.2%)	6 (33.4%)	13 (54.2%)	0.104
Clinical research experience	34 (81.0%)	12 (66.7%)	22 (91.7%)	0.041
Experience in lung transplantation anesthesia				0.602
Yes	9 (21.4%)	4 (22.2%)	5 (20.8%)	
No	33 (78.6%)	14 (77.8%)	19 (79.2%)	
Experience in cardiac anesthesia				0.760
Yes	39 (92.9%)	16 (88.9%)	23 (95.8%)	
No	3 (7.1%)	2 (11.1%)	1 (4.2%)	
Experience with Swan-Ganz catheterization				0.757
Yes	31 (73.8%)	13 (72.2%)	18 (75.0%)	
No	11 (26.2%)	5 (27.8%)	6 (25.0%)	
Experience with TEE				0.670
Yes	24 (57.1%)	11 (61.1%)	13 (54.2%)	
No	18 (42.9%)	7 (38.9%)	11 (45.8%)	
Duration of training (months)				0.312
<1	8 (19.0%)	4 (22.2%)	4 (16.7%)	
1–3	24 (57.1%)	7 (38.9%)	17 (70.8%)	
4–6	10 (23.8%)	7 (38.9%)	3 (12.5%)	
Number of lung transplantation anesthesia cases involved in during training				0.840
≤5	9 (21.4%)	4 (22.2%)	5 (20.8%)	
6–10	5 (11.9%)	2 (11.1%)	3 (12.5%)	
11–20	12 (28.6%)	7 (38.9%)	5 (20.8%)	
21-30	10 (23.8%)	3 (16.7%)	7 (29.2%)	
>30	6 (14.3%)	3 (16.7%)	3 (12.5%)	
Opinion on duration of training				0.706
Too short	8 (19.0%)	4 (22.2%)	4 (16.7%)	
Suitable	34 (81.0%)	14 (77.8%)	20 (83.3%)	
Too long	0 (0%)	0 (0%)	0 (0%)	
Desired duration of training (months)				0.044
≤1	4 (9.5%)	2 (11.1%)	2 (8.3%)	
1–3	24 (57.1%)	6 (33.3%)	18 (75.0%)	
4–6	13 (31.0%)	9 (50%)	4 (16.7%)	
≥6	1 (2.4%)	1 (5.6%)	0 (0%)	
Opinion of work intensity				0.125
Very high	4 (9.5%)	0 (0%)	4 (16.7%)	
High	16 (38.1%)	7 (38.9%)	9 (37.5%)	
Moderate	22 (52.4%)	11 (61.1%)	11 (45.8%)	
Low	0 (0%)	0 (0%)	0 (0%)	
Very low	0 (0%)	0 (0%)	0 (0%)	
Level of satisfaction with training				0.129
Very high	20 (47.6%)	11 (61.1%)	9 (37.5%)	
High	22 (52.4%)	7 (38.9%)	15 (62.5%)	
Moderate	0 (0%)	0 (0%)	0 (0%)	
Low	0 (0%)	0 (0%)	0 (0%)	
Very low	0 (0%)	0 (0%)	0 (0%)	
Weaknesses of training				
Short duration	4 (9.5%)	2 (11.1%)	2 (8.3%)	0.580
Lack of theoretical courses	29 (69.0%)	11 (61.1%)	18 (75.0%)	0.335
Lack of scenario simulation teaching	23 (54.8%)	9 (50.0%)	14 (58.3%)	0.591
Few lung transplant anesthesia opportunities	10 (23.8%)	4 (22.2%)	6 (25.0%)	0.566
Few clinical procedures opportunities	2 (4.8%)	1 (5.6%)	1 (4.2%)	0.679
Others	6 (14.3%)	4 (22.2%)	2 (8.3%)	0.204
Desired program				
TEE	30 (71.4%)	11 (61.1%)	19 (79.2%)	0.200
Ultrasound-guided nerve block/puncture	6 (14.3%)	3 (16.7%)	3 (12.5%)	0.519
Postoperative analgesia	4 (9.5%)	3 (16.7%)	1 (4.2%)	0.202
Swan-Ganz catheter	17 (40.5%)	9 (50.0%)	8 (33.3%)	0.276
One-lung ventilation	4 (9.5%)	3 (16.7%)	1 (4.2%)	0.202
ECMO	27 (64.3%)	14 (77.8%)	13 (54.2%)	0.114
Others	5 (11.9%)	2 (11.1%)	3 (12.5%)	0.639

TEE, transesophageal echocardiography; ECMO, extracorporeal membrane oxygenation.

Most doctors’ (57.1%, n = 24) training duration was 1–3 months. All (100%, n = 42) doctors were highly or very highly satisfied with the training and there was no significant difference in level of satisfaction with the training between the two groups of doctors. Eight (19.0%) doctors considered the training too short and the remainder considered it suitable. Nine (50%) intermediate-level doctors desired a training period of 4–6 months, while 18 (75%) in senior-level positions desired a duration of 1–3 months (P = 0.044). Twenty-two (52.4%) doctors considered the work intensity moderate, and 16 (38.1%) considered it high. Regarding the shortfalls in training, most doctors reported a lack of theoretical courses (69.0%, n = 29), followed by a lack of scenario simulation teaching (54.8%, n = 23), and limited cases of lung transplant anesthesia participated (23.8%, n = 10). During the training period, the programs commonly desired by trainee anesthesiologists were TEE (71.4%, n = 30), extracorporeal membrane oxygenation (ECMO, 64.3%, n = 27) and Swan-Ganz catheterization (40.5%, n = 17).

### 3.2 Current status in trainee anesthesiologists’ hospitals

Some information about trainee anesthesiologists' hospitals is shown in Table 2, such as the daily number of surgeries, number of beds, and number of pulmonary and cardiac surgeries per year. In 2020, more than half of trainee anesthesiologists (52.4%, n = 22) said that their hospitals had not performed lung transplants. Forty (95.2%) doctors had ECMO technology and thirty-eight (90.5%) had cardiopulmonary bypass (CPB) technology in their hospitals. The most common department which took charge of perioperative ECMO in trainee anesthesiologists' hospitals was the ICU (45.2%, n = 19), followed by the ECMO unit (31.0%, n = 13) and thoracic surgery (16%, n = 7). In trainee anesthesiologists’ hospitals, CPB unit (52.4%, n = 22) was the most common department in charge of perioperative CPB, followed by anesthesiology (23.8%, n = 10) and thoracic surgery (14.3%, n = 6).

**Table 2:** Current status of trainee doctors’ hospitals.

	All doctors (n = 42)	Intermediate (n = 18)	Senior (n = 24)	P-value
Number of daily surgeries				0.001
≤50	6 (14.3%)	1 (5.6%)	5 (20.8%)	
51–100	4 (9.5%)	4 (22.0%)	0 (0.0%)	
101–150	7 (16.7%)	2 (11.1%)	5 (20.8%)	
151–200	10 (23.8%)	1 (5.6%)	9 (37.5%)	
201–250	4 (9.5%)	4 (22.2%)	0 (0.0%)	
>250	11 (26.5%)	6 (33.3%)	5 (20.8%)	
Number of beds				0.913
≤1000	4 (9.5%)	2 (11.1%)	2 (8.3%)	
1001–2000	5 (11.9%)	2 (11.1%)	3 (12.5%)	
2001–3000	13 (31.0%)	5 (27.8%)	8 (33.3%)	
3001–4000	11 (26.2%)	4 (22.2%)	7 (29.2%)	
>4000	9 (21.4%)	5 (27.8%)	4 (16.7%)	
Number of pulmonary surgeries per year				0.982
≤500	10 (23.8%)	5 (27.8%)	5 (20.8%)	
501–1500	8 (19.0%)	3 (16.7%)	5 (20.8%)	
1501–2500	8 (19.0%)	3 (16.7%)	5 (20.8%)	
2501–3500	7 (16.7%)	3 (16.7%)	4 (16.7%)	
>3500	9 (21.4%)	4 (22.2%)	5 (20.8%)	
Number of cardiac surgeries per year				0.261
≤100	11 (26.2%)	6 (33.3%)	5 (20.8%)	
101–500	10 (23.8%)	3 (16.7%)	7 (29.2%)	
501–1000	3 (7.1%)	0 (0.0%)	3 (12.5%)	
1001–1500	7 (16.7%)	4 (22.2%)	3 (12.5%)	
>1500	11 (26.2%)	5 (27.8%)	6 (25.0%)	
Number of lung transplantation surgeries per year				0.198
0	22 (52.4%)	9 (50.0%)	14 (58.4%)	
1–5	10 (23.8%)	6 (33.3%)	4 (16.7%)	
6–10	3 (7.1%)	0 (0.0%)	3 (12.5%)	
11–30	1 (2.4%)	0 (0.0%)	1 (4.2%)	
31–50	1 (2.4%)	1 (5.6%)	0 (0.0%)	
>50	4 (9.5%)	2 (11.1%)	2 (8.3%)	
Owning ECMO technology				0.321
Yes	40 (95.2%)	18 (100%)	22 (91.7%)	
No	2 (4.8%)	0 (0.0%)	2 (8.3%)	
Department in charge of perioperative ECMO				0.148
Anesthesiology	1 (2.4%)	1 (5.6%)	0 (0.0%)	
Thoracic surgery	7 (16.7%)	3 (16.7%)	4 (16.7%)	
ICU	19 (45.2%)	6 (33.3%)	13 (54.2%)	
ECMO unit	13 (31.0%)	8 (44.4%)	5 (20.8%)	
No ECMO	2 (4.8%)	0 (0.0%)	2 (8.3%)	
Owning CPB technology				0.095
Yes	38 (90.5%)	18 (100.0%)	20 (83.3%)	
No	4 (9.5%)	0 (0.0%)	4 (16.7%)	
Department in charge of perioperative CPB				0.004
Anesthesiology	10 (23.8%)	6 (33.3%)	4 (16.7%)	
Thoracic surgery	6 (14.3%)	0 (0.0%)	6 (25.0%)	
ICU	0 (0.0%)	0 (0.0%)	0 (0.0%)	
CPB unit	22 (52.4%)	12 (66.7%)	10 (41.7%)	
No CPB	4 (9.5%)	0 (0.0%)	4 (16.7%)	
Hemodynamic monitoring preferred during lung transplant surgery				
Swan-Ganz catheterization	38 (90.5%)	18 (100.0%)	20 (83.3%)	0.095
Calibrated pulse contour analysis	35 (83.3%)	12 (66.7%)	23 (95.8%)	0.018
Uncalibrated pulse contour analysis	33 (78.6%)	15 (83.3%)	18 (75.0%)	0.398
TEE	23 (54.8%)	13 (72.2%)	10 (41.7%)	0.049
Others	3 (7.1%)	1 (5.6%)	2 (8.3%)	0.609
DCD department				0.481
Yes	36 (85.7%)	16 (88.9%)	20 (83.3%)	
No	6 (14.3%)	2 (11.1%)	4 (16.7%)	
Numbers of trainee anesthesiologists per year				0.411
≤10	19 (45.2%)	7 (38.9%)	12 (50.0%)	
11–30	14 (33.3%)	5 (27.8%)	9 (37.5%)	
31–50	5 (11.9%)	3 (16.7%)	2 (8.3%)	
>50	4 (9.5%)	3 (16.7%)	1 (4.2%)	
Staff responsible for trainee anesthesiologists				0.609
Yes	39 (92.9%)	17 (94.4%)	22 (91.7%)	
No	3 (7.1%)	1 (5.6%)	2 (8.3%)	
Teaching program for trainee anesthesiologists				0.321
Yes	40 (95.2%)	18 (100%)	22 (91.7%)	
No	2 (4.8%)	0 (0.0%)	2 (8.3%)	
Certification evaluation for trainee anesthesiologists				
Theoretical tests	29 (69.0%)	11 (61.1%)	18 (75.0%)	0.335
Skills evaluation	25 (59.5%)	8 (44.4%)	17 (70.8%)	0.085
Workload evaluation	14 (33.3%)	4 (22.2%)	10 (41.7%)	0.186
None	9 (21.4%)	4 (22.2%)	5 (20.8%)	0.602

ECMO, extracorporeal membrane oxygenation; CPB, cardiopulmonary bypass; DCD, Donation after Cardiac Death.

For hemodynamic monitoring during lung transplant surgery, Swan-Ganz catheterization (90.5%, n = 38), calibrated pulse contour analysis (83.3%, n = 35), uncalibrated pulse contour analysis (78.6%, n = 33) and TEE (54.8%, n = 23) were preferred. Significantly more senior doctors chose calibrated pulse contour analysis (P = 0.018) and significantly more intermediate ones choosing TEE (P = 0.049).

Thirty-six (85.7%) doctors said their hospitals had a Donation after Cardiac Death (DCD) department. Thirty-nine (92.9%) doctors said their hospitals had staff responsible for trainee anesthesiologists, and forty (95.2%) said there were teaching programs for trainee anesthesiologists. Regarding the certification evaluation for trainee anesthesiologists, twenty-nine (69.0%) doctors reported that their hospitals used theoretical tests, twenty-five (59.5%) reported that their hospitals used skills evaluation, and fourteen (33.3%) reported that their hospitals used workload evaluation, while nine (21.4%) reported no certification evaluation for trainee anesthesiologists in their hospitals.

## 4 Discussion

The development of lung transplantation is progressing with each passing day [5, 6]. Anesthesiologists play an important role in every stage of the perioperative period of lung transplantation, such as induction of anesthesia, lung dissection, one-lung ventilation, pulmonary vascular occlusion, mechanical circulatory support, reperfusion, chest closure and in the immediate postoperative period [7, 8]. A recent survey noted that the perioperative management of anesthesiologists affected recipients’ outcomes [4, 8]. The intraoperative management of lung transplantation is a challenging issue for anesthesiologists [9, 10]. In this study, most of the trainee anesthesiologists had experience in cardiothoracic anesthesia. It indicated that hospitals in China that perform or are preparing to perform lung transplantation were more willing to send doctors with rich experience in cardiothoracic anesthesia to study lung transplantation anesthesia. Our study revealed that senior doctors had more clinical research experience than did intermediate ones, with no statistically significant difference between the two groups in basic research experience. Young anesthesiologists in China might lack training in clinical research. A recent study suggested that the quality of clinical training for Chinese MD graduates should be improved [11]. Clinical training does not only entail clinical practice, but also clinical research which is often neglected.

In this study, more than half of the trainee anesthesiologists’ duration of training lasted 1–3 months which most of the doctors found suitable. Doctors in intermediate-level positions preferred a longer training period (4–6 months), while seniors preferred a shorter training period (1–3 months). Compared with intermediate-level doctors, senior doctors tended to have more clinical experience and theoretical knowledge and learned faster, hence, they might require less time to train. This suggests that the duration of training may need to be different depending on the seniority of the doctors. According to the volume of lung transplant operations in our center, we suggest that intermediate-level trainee anesthesiologists conduct at least 6 months of training with more than 20 cases, and senior trainee anesthesiologists conduct at least 3 months of training with more than 10 cases, in order to meet the minimum training requirements. Nearly half of the doctors had very high levels of satisfaction with training and the other half had high levels of satisfaction. Over half of the doctors said that the work intensity during training was moderate. This confirmed our training in lung transplantation anesthesia is suitable and satisfactory to trainees.

Although all of the trainee anesthesiologists came from some of the top hospitals in China with solid theoretical foundation and rich clinical experience, most of both the senior- and intermediate-level doctors considered the lack of theoretical courses and the lack of scenario simulation teaching (SST) as major deficiencies in training. In recent years, with the development of lung transplantation anesthesia, systematic theory and practice standards have been established [7, 10, 12]. A systematic anesthesia theory course for lung transplantation in the context of China is urgently needed, which is what we are focusing on. SST uses various simulation methods to reproduce clinical case, which can provide learners with the conditions and environment to acquire clinical knowledge and skills without adding risk to the patient, and can improve the enthusiasm and engagement of students [13, 14, 15]. Simulation teaching has played an important role in the training of anesthesia residents [16, 17]. It not only helps anesthesiologists improve their basic theoretical knowledge, but also has a more significant impact on skills training and perioperative crisis management [17, 18, 19, 20]. In the training of thoracic anesthesia, the application of SST may also act as a method to improve the impact of teaching [21]. However, the management of lung transplantation anesthesia is very complicated, and organizing and implementing simulation teaching is difficult. A problem facing current teaching and which requires consideration is identifying means of maximizing the impact of teaching using limited resources [22].

This study showed that the most desirable program was TEE, which is a technique of great value in the diagnosis and treatment of cardiac function, structure, and preload during lung transplantation [10, 12, 23, 24]. A growing number of anesthesiologists routinely use TEE monitoring during lung transplantation anesthesia [8, 9, 25, 26]. However, the use of TEE in China is disappointingly low. Nearly half of the anesthesiologists in the study had no TEE experience. Therefore, the training of TEE use in lung transplantation anesthesia is imperative. TEE use has a long learning curve. Although a large number of books, videos, and lectures are available for learning, the training in the use of TEE still relies heavily on hands-on training in the clinical setting or using a TEE simulator [27, 28, 29]. TEE training has always been difficult in anesthesiology teaching. According to the results of this survey, intermediate-level doctors were more willing to use TEE in lung transplantation. It demonstrated that TEE technology is more accepted by young anesthesiologists and has better development prospects in the future. Increasing the content of TEE training and establishing the use of TEE as a standard in lung transplantation anesthesia is important.

The second most desired topic was the use of ECMO. In recent years, the use of ECMO support in lung transplantation has become a trend. As a bridging therapy for the safety of lung transplantation in patients with end-stage lung disease, ECMO can optimize patients' preoperative ventilation requirements, stabilize and improve end-stage organ perfusion, increase the possibility of critically-ill patients receiving lung transplantation, and minimize the potentially life-threatening factors pre-transplantation, such as breathing and circulatory instability [30, 31, 32]. Some recent studies have indicated that the planned utilization of ECMO was associated with reductions in mortality rates in critically-ill patients on the waiting list, intubation time, length of hospital stand and early mortality after lung transplantation. Furthermore, the planned use of ECMO decreased the risk of primary graft dysfunction and bleeding complications [33, 34, 35]. The widespread use of ECMO support affects requirements for anesthesia management in lung transplantation [36]. This survey found that almost all hospitals where the trainee anesthesiologists work had ECMO technology, but only a small number of hospitals had ECMO managed by anesthesiologists. ECMO-related knowledge and technology might be the weakness of Chinese anesthesiologists. We should be more dedicated to include ECMO training in the future teaching of lung transplantation anesthesia.

Due to the uneven distribution of medical resources in China, it was an efficient and convenient way for most hospitals in China to improve the technical level of a certain professional field by sending technical backbones to relevant medical centers for further study. Subsequently, major hospitals attached foremost importance to the training of specialist doctors. Nearly all of the participating doctors reported that their departments had staff who were part of the teaching program for trainee anesthesiologists. However, some hospitals lacked the quality control of the training of specialist doctors. More than 20% of the doctors in this survey indicated that their hospitals did not have certification evaluation for trainee anesthesiologists. In the training of anesthesiologists, the completion of an assessment is an important means to assess the quality of training, not only with regards to the theoretical knowledge assessment, but also the professional skills assessment [37]. In the United States, training in anesthesia for lung transplantation is believed not to have kept up with developments in the field of lung transplantation. So in 2020, The Society for the Advancement of Anesthesia (SATA) proposed a white paper for anesthesia training in this area. This white paper suggested six core competencies in lung transplant anesthesia training: Practice-Based Learning, Professionalism, Interpersonal and Communication Skills, Systems-Based Practice, Medical Knowledge, and Patient Care and Procedural Skills [38]. In China, the training of anesthesia for lung transplantation has a long way to go. Most hospitals that perform lung transplants did not have systematic training due to the small number of operations. They had to send their anesthesiologists to several top centers for training. Even in top centers, training courses and evaluation systems for lung transplant anesthesia need to be improved. Ours is the largest lung transplant center and training center for lung transplant medical staff in China [2]. It is especially important to establish a scientific and complete method of evaluating trainee anesthesiologists who have completed training in lung transplantation anesthesia.

This study had several limitations. First, the survey was performed at a single training hospital in China. Second, the time for each trainee doctor's visit was unequal, and our training course might also be different at the time of their visit. Third, the sample size is small, we may need to more trainee anesthesiologists in China to be sure.

## 5 Conclusions

We should set up different training programs according to the training needs and seniority of the trainee anesthesiologists and consider adding theoretical courses and scenario simulation training to improve its impact. Moreover, we should also pay greater attention to the training of TEE use and ECMO-related content. Finally, we should establish a standardized scientific completion assessment system for trainee anesthesiologists.

## Declarations

### Author contribution statement

Yan Zhou: Conceived and designed the experiments; Performed the experiments; Analyzed and interpreted the data; Contributed survey, analysis tools or data; Wrote the paper.

Zhong Qin: Performed the experiments; Analyzed and interpreted the data; Contributed survey, analysis tools or data.

Guilong Wang and Wenyi Chen: Performed the experiments.

Xin Zhang: Conceived and designed the experiments; Contributed survey, analysis tools or data; Wrote the paper.

### Funding statement

Dr. Xin Zhang was supported by major scientific research projects from Municipal Health Commission of Wuxi [Z202101], guiding project from Science and Technology Bureau of Wuxi [NZ2021002], National Natural Science Foundation of China [82271251]. Dr. Guilong Wang was supported by Advanced Technique Promotion Program from Municipal Health Commission of Wuxi [T202202].

### Data availability statement

Data included in article/supp. material/referenced in article.

### Declaration of interest's statement

The authors declare no competing interests.

### Additional information

Supplementary content related to this article has been published online at https://doi.org/10.1016/j.heliyon.2022.e12428.

## Appendix A Supplementary data

Questionnaire.docx Questionnaire.docx
