# Supplementary material for: Current status and training needs of trainee anesthesiologists in lung transplantation anesthesia in China: A single-center survey
Source: Heliyon. 2022 Dec 19;8(12):e12428. doi: 10.1016/j.heliyon.2022.e12428 (PMC9800522; doi:10.1016/j.heliyon.2022.e12428)
Supplement: Questionnaire.docx [file mmc1.docx]

Questionnaire on anesthesia training of lung transplantation in XXX.

1. What is your sex?
   1. Male
   2. Female
2. What is your age? (year)
   1. ＜30
   2. 30-35
   3. 36-40
   4. 41-45
   5. ＞45
3. What is your highest level of education?
   1. Bachelor’s Degree
   2. Master Degree
   3. PHD Degree
4. What is your professional title?
   1. Doctor in charge
   2. Associate senior doctor
   3. Senior doctor
5. What are your years of work in anesthesiology department?
   1. ＜5
   2. 5-10
   3. 10-15
   4. ＞15
6. What is your research experience? (Multiple-choice)
   1. Basic research experience
   2. Clinical research experience
   3. Neither
7. Do you have experience in lung transplantation anesthesia?
   1. Yes
   2. No
8. Do you have experience in cardiac anesthesia?
   1. Yes
   2. No
9. Do you have experience with Swan-Ganz catheter?
   1. Yes
   2. No
10. Do you have experience with TEE?
    1. Yes
    2. No
11. What is your duration of training? (month)
    1. ＜1
    2. 1-3
    3. 4-6
12. What is your number of lung transplantation anesthesia cases involved during training?
    1. ≤5
    2. 6-10
    3. 11-20
    4. 21-30
    5. ＞30
13. What is your opinion on duration of training
    1. Too short
    2. Suitable
    3. Too long
14. What is your desired duration of training? (month)
    1. ≤1
    2. 1-3
    3. 4-6
    4. ≥6
15. What is your opinion of working intensity?
    1. Very high
    2. High
    3. Moderate
    4. Low
    5. Very low
16. What is your satisfaction with training?
    1. Very high
    2. High
    3. Moderate
    4. Low
    5. Very low
17. What is the weakness of current training? (Multiple-choice)
    1. Short duration
    2. Lack of theoretical courses
    3. Lack of scenario simulation teaching
    4. Low lung transplant anesthesia opportunity
    5. Low clinical procedures opportunity
    6. Others
18. What is your desired program? (Multiple-choice)
    1. TEE
    2. Ultrasound block/punction
    3. Postoperative analgesia
    4. SwanGanz catheter
    5. One-lung ventilation
    6. ECMO
    7. Others
19. What is the daily surgery amount in your hospital?
    1. ≤50
    2. 51-100
    3. 101-150
    4. 151-200
    5. 201-250
    6. ＞250
20. What is the number of beds in your hospital?
    1. ≤1000
    2. 1001-2000
    3. 2001-3000
    4. 3001-4000
    5. ＞4000
21. What is the pulmonary surgery amount per year in your hospital?
    1. ≤500
    2. 501-1500
    3. 1501-2500
    4. 2501-3500
    5. ＞3500
22. What is the cardiac surgery amount per year in your hospital?
    1. ≤100
    2. 101-500
    3. 501-1000
    4. 1001-1500
    5. ＞1500
23. What is the lung transplantation surgery amount in recent year in your hospital?
    1. 0
    2. 1-5
    3. 6-10
    4. 11-30
    5. 31-50
    6. ＞50
24. Does your hospital own ECMO technology?
    1. Yes
    2. No
25. Which department is in charge of perioperative ECMO in your hospital?
    1. Anesthesiology
    2. Thoracic surgery
    3. ICU
    4. ECMO unit
    5. No ECMO
26. Does your hospital own CPB technology?
    1. Yes
    2. No
27. Which department is in charge of perioperative CPB in your hospital?
    1. Anesthesiology
    2. Thoracic surgery
    3. ICU
    4. CPB unit
    5. No CPB
28. What hemodynamic monitoring do you prefer during lung transplant surgery? (Multiple-choice)
    1. SwanGanz catheter
    2. Calibrated pulse contour analysis
    3. Uncalibrated pulse contour analysis
    4. TEE
    5. Others
29. Does your hospital have DCD department?
    1. Yes
    2. No
30. What are the numbers of training anesthesiologists per year in your hospital?
    1. ≤10
    2. 11-30
    3. 31-50
    4. ＞50
31. Does your hospital have staff responsible for training anesthesiologists?
    1. Yes
    2. No
32. Does your hospital have teaching program for training anesthesiologists?
    1. Yes
    2. No
33. What is the certification evaluation for training anesthesiologists in your hospital? (Multiple-choice)
    1. Theoretical tests
    2. Skill evaluation
    3. Workload evaluation
    4. None
